# Pressure‐Induced Formation and Mechanical Properties of 2D Diamond Boron Nitride

**DOI:** 10.1002/advs.202002541

**Published:** 2020-12-11

**Authors:** Filippo Cellini, Francesco Lavini, Elton Chen, Angelo Bongiorno, Filip Popovic, Ryan L. Hartman, Remi Dingreville, Elisa Riedo

**Affiliations:** ^1^ Tandon School of Engineering New York University Brooklyn NY 11201 USA; ^2^ Department of Physics New York University New York NY 10003 USA; ^3^ Center for Integrated Nanotechnologies Sandia National Laboratories Albuquerque NM 87185 USA; ^4^ Department of Chemistry College of Staten Island City University of New York Staten Island NY 10314 USA; ^5^ CUNY Graduate Center Ph.D. Program in Chemistry and Physics New York NY 10016 USA

**Keywords:** h‐BN, mechanical properties, molecular dynamics, nanoindentation, phase transitions

## Abstract

Understanding phase transformations in 2D materials can unlock unprecedented developments in nanotechnology, since their unique properties can be dramatically modified by external fields that control the phase change. Here, experiments and simulations are used to investigate the mechanical properties of a 2D diamond boron nitride (BN) phase induced by applying local pressure on atomically thin h‐BN on a SiO_2_ substrate, at room temperature, and without chemical functionalization. Molecular dynamics (MD) simulations show a metastable local rearrangement of the h‐BN atoms into diamond crystal clusters when increasing the indentation pressure. Raman spectroscopy experiments confirm the presence of a pressure‐induced cubic BN phase, and its metastability upon release of pressure. Å‐indentation experiments and simulations show that at pressures of 2–4 GPa, the indentation stiffness of monolayer h‐BN on SiO_2_ is the same of bare SiO_2_, whereas for two‐ and three‐layer‐thick h‐BN on SiO_2_ the stiffness increases of up to 50% compared to bare SiO_2_, and then it decreases when increasing the number of layers. Up to 4 GPa, the reduced strain in the layers closer to the substrate decreases the probability of the sp^2^‐to‐sp^3^ phase transition, explaining the lower stiffness observed in thicker h‐BN.

## Graphical abstract

Novel 2D materials hold promising prospects for groundbreaking progress in mechanical technologies. Indentation experiments and molecular dynamics simulations demonstrate that under pressure few‐layer hexagonal boron nitride (BN) on a SiO_2_ substrate undergoes a structural sp^2^‐to‐sp^3^ phase transformation to a 2D Diamond BN structure, that features up to 50% increase in stiffness compared to the bare substrate.

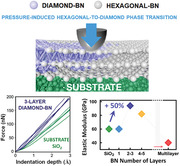

Among the class of 2D materials, atomically thin hexagonal boron nitride (h‐BN) shares several similarities with graphene, including the crystal structure, and excellent mechanical strength.^[^ ^1^ ^]^ Nevertheless, h‐BN exhibits many distinct properties that make it preferable to graphene in some applications.^[^ ^2^ ^]^ As a flat insulator with wide bandgap, h‐BN can be used as dielectric substrate for graphene and other 2D materials‐based electronic devices.^[^ ^3^, ^4^ ^]^ Its outstanding chemical stability and resistance to oxidation even at high temperatures,^[^ ^5^, ^6^ ^]^ together with its superior thermal expansion coefficient^[^ ^7^ ^]^ and high thermal conductivity,^[^ ^8^ ^]^ make h‐BN one of the best electrically insulating thermal conductors. h‐BN is also an ideal candidate for protective coatings,^[^ ^9^, ^10^ ^]^ deep‐UV light^[^ ^11^ ^]^ emitters, and single‐photon quantum sources.^[^ ^12^ ^]^ Similarly to other 2D materials,^[^ ^4^, ^13^ ^]^ boron nitride exists in different polytypes, such as the sp^2^ hexagonal structure, which is equivalent to graphite, and the sp^3^ cubic (c‐BN) diamond phase.^[^ ^14^ ^]^ The ability to induce and control the transformation between the various crystalline structures is of key importance in the framework of tailoring the mechanical and physical properties of 2D materials.^[^ ^15^, ^16^, ^17^, ^18^, ^19^ ^]^ Analogously to graphite and diamond, bulk h‐BN is well known to undergo a rearrangement of chemical bonds with consequent formation of sp^3^‐hybridized structures upon a combination of high pressure and temperature.^[^ ^20^, ^21^, ^22^ ^]^ Recent density functional theory (DFT) simulations^[^ ^23^ ^]^ have reported on the pressure induced transformation of few‐layer h‐BN into different types of sp^3^ BN structures. Other simulations^[^ ^24^ ^]^ have shown a pressure induced formation of a conductive structure (called “bonitrol”) when few‐layer h‐BN is in presence of water, and experiments^[^ ^24^ ^]^ supported these findings by monitoring the change in work function of few‐layer h‐BN upon local compression in a humid environment. However, so far there has been no direct experimental evidence of a pressure‐induced conversion of atomically thin h‐BN into sp^3^ BN structures, and no experiments have investigated the mechanical properties of the pressure induced diamond BN phase.

Very recently, the atomic force microscopy (AFM) based modulated nano/Å‐indentation (MoNI/ÅI)^[^ ^15^, ^25^, ^26^, ^27^ ^]^ technique has been shown to be an excellent method to study the indentation stiffness of ultrahard atomically thin films on rigid substrates. Here, MoNI/ÅI combined with large scale MD simulations are used to study the pressure induced phase transition of few‐layer h‐BN into sp^3^‐diamond BN, and to investigate the associated change in elastic properties as a function of the number of atomic layers. MoNI/ÅI experiments and MD indentation simulations are performed on h‐BN flakes of various thickness, exfoliated and supported on a silicon oxide (SiO_2_) substrate. Both measurements and theory find that at pressures of about 2–4 GPa the indentation stiffness of monolayer h‐BN on SiO_2_ is the same of the bare SiO_2_ substrate, whereas it is larger and has a maximum for 2‐ and 3‐layer h‐BN, with an increase of up to 50% compared to bare SiO_2_, and then it decreases when increasing the number of layers. For pressures up to 4 GPa, this decrease can be understood in terms of a reduced strain in the layers closer to the substrate when the h‐BN film is thicker, therefore decreasing the probability of the sp^2^‐to‐sp^3^ phase transition in the underlying layers. It is noteworthy the fact that the stiffening phenomenon, being related to the nucleation and growth of the sp^3^‐phase in the different atomic layers, is a stochastic effect, as highlighted in both experiments and simulations. Finally, we perform Kelvin probe force microscopy (KPFM) and Raman spectroscopy experiments on regions of the h‐BN bilayer flakes that have been previously compressed to investigate the metastability and structure of the induced sp^3^ phase. Raman experiments indicate that the new sp^3^ phase is indeed a cubic BN phase, and it is stable for about an hour after release of pressure.

The h‐BN samples are prepared by mechanical exfoliation of bulk crystals on a SiO_2_ substrate surface following the procedure described in the Experimental Section. Several h‐BN flakes are isolated and their morphology, and in particular the number of layers in each region of the flake, is assessed using optical microscopy and AFM topography imaging, see Figure S1 in the Supporting Information. Recent experiments have demonstrated the formation of ultrahard, ultrastiff sp^3^ monolayer diamond, named “diamene”, when locally pressurizing epitaxial graphene films at room temperature during MoNI/ÅI testing.^[^ ^15^, ^25^, ^26^ ^]^ Experiments and theory indicate that the indentation modulus of diamene is similar to that of bulk diamond. Similarly, here the nanomechanical behavior of atomically thin h‐BN flakes is probed using MoNI/ÅI, which allows to obtain sub‐Ångstrom resolution indentation curves.^[^ ^26^ ^]^ During the MoNI/ÅI experiments, conducted at room temperature, an AFM diamond conical indenter (indenter radius ≈ 100 nm) is first brought into contact with the h‐BN flake at the desired maximum normal load. It is then slowly retracted from the sample surface while the normal load is gradually reduced until the indenter loses contact, as displayed in **Figure** **1**a. The force versus indentation depth curve is therefore acquired during the unloading phase, as extensively discussed in Ref. [26]. Experimental details regarding the MoNI/ÅI curves can be found in the Experimental Section. Leveraging the imaging capabilities of the integrated AFM, the MoNI/ÅI curves are selectively acquired on specific points of interest on the h‐BN flakes and on the bare SiO_2_ substrate. This allows a direct comparison of the stiffness of the bare substrate with that of h‐BN flakes with different number of layers.

**Figure 1:**
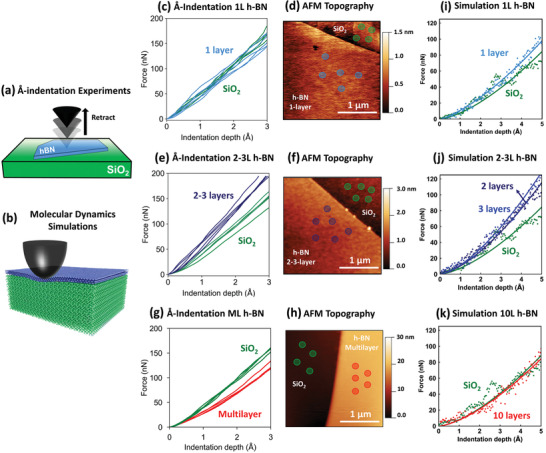
a) Schematic of the MoNI/ÅI experiments performed on h‐BN flakes. b) Schematic of the Molecular Dynamics model simulating indentation of a spherical rigid indenter pressing down on a h‐BN film (here, 3‐layer h‐BN). c) Experimental MoNI/ÅI curves measured on 1‐layer (1L) h‐BN flake and SiO_2_, e) 2‐3‐layer (2‐3L) h‐BN flake and SiO_2_, and g) multilayer (ML) h‐BN flake and SiO_2_. d) AFM topography image of the areas where the indentation curves are collected, respectively for 1L, f) 2–3L, h) ML, and SiO_2_. The color scheme used for the markers is the same as in the MoNI/ÅI plots. i) Force‐indentation curves extracted from the MD indentations calculated on 1‐layer h‐BN film, j) on 2‐layer and 3‐layer h‐BN film, and k) on 10‐layer h‐BN film, each one compared to that calculated on SiO_2_ substrate.

Figure 1c displays the experimental MoNI/ÅI curves measured on 1‐layer h‐BN and on the bare SiO_2_ substrate, while Figure 1d displays the AFM topography of the tested flake, and the exact positions on the surface area where the indentations are performed. As expected, the indentation curves measured on 1‐layer (1L) h‐BN on SiO_2_ are identical to the curves measured on bare SiO_2_. **Figure** **2**e displays the indentation curves measured on 2‐ and 3‐layer (2L and 3L) h‐BN flakes, and on the bare SiO_2_ substrate. The topography of the h‐BN flake is presented in Figure 2f, together with the positions where the indentations are performed. We group together data from 2L and 3L h‐BN because it is generally challenging to distinguish with absolute certainty between 2L and 3L h‐BN flakes by just referring to the AFM topography profiles (see Figure S1 in the Supporting Information) and also because, when a clear distinction is detectable, the curves extracted from 2L areas show on average the same mechanical response as those extracted from 3L h‐BN (see Figure S2 in the Supporting Information). Differently from 1L flakes, 2L and 3L h‐BN indentation curves show a significantly steeper slope than the bare SiO_2_, indicating that 2L and 3L h‐BN is much stiffer than the SiO_2_ substrate. This result is quite surprising since the transverse Young's modulus of bulk h‐BN is ≈32 GPa^[^ ^28^ ^]^ (similar to graphite), and previous observations on graphene flakes mechanically exfoliated on SiO_2_, indicate that the indentation stiffness of 2L and 3L graphene flakes is generally smaller than that of the bare SiO_2_ substrate, and progressively decreases with increasing number of layers.^[^ ^27^ ^]^ In the case of h‐BN flakes, we observe a maximum in indentation stiffness for 2–3L, whereas the flakes/SiO_2_ system becomes softer and softer for flakes with L > 3. Figure 1g shows the curves measured on multilayer (ML) h‐BN (>20 layers) on SiO_2_, which indicate that the ML h‐BN/SiO_2_ system is much softer than bare SiO_2_ for these indentations depths. These results suggest that, similarly to the epitaxial graphene case,^[^ ^15^ ^]^ a pressure induce sp^2^‐to‐sp^3^ phase transition may occur in exfoliated h‐BN atomically thin crystals, giving rise to a large increase in indentation stiffness.

**Figure 2:**
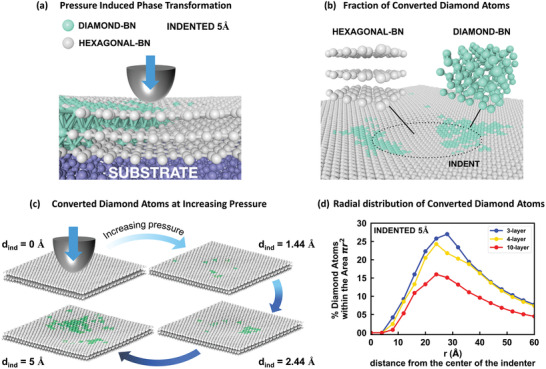
a) Illustrations of lattice in a 3‐layer h‐BN sheet on SiO_2_ substrate undergoing the pressure‐induced phase transition to diamond BN structure upon indentation. b) Snapshot of the MD indentation of the 3‐layer h‐BN film (indentation depth *d*_ind_ = 5 Å), illustrating the crystallographic arrangement of the BN atoms in the area around the indent. c) Evolution of the pressure‐induced phase transformation of 3‐layer h‐BN film under increasing indentation pressure, as simulated by MD calculations. The atoms color scheme is the same as in (a). d) Radial distribution of the relative amount of BN atoms per volume converted to diamond BN in 3‐layer, 4‐layer, and 10‐layer h‐BN films, for indentation depth *d*_ind_ = 5Å.

To shed light into the origin of the measured increase in indentation stiffness for 2–3L h‐BN on SiO_2_, and understand the relationship between this increase and a possible pressure induced sp^2^‐to‐sp^3^ phase transition, we carry out large scale molecular dynamics (MD) calculations simulating the indentation in h‐BN films of different thickness on SiO_2_, see Figure 1b. Details regarding the MD modeling used here can be found in the Experimental Section. In the MD simulations, a spherical indenter with a radius, *R*_ind_, of 10 nm is used to reproduce the action of the AFM indenter in the MoNI/ÅI experiments. The MD simulated indentation curves on bare SiO_2_, 1L, 2L–3L, and 10L h‐BN are displayed in Figure 1h,j,k, respectively, and show excellent agreement with the respective MoNI/ÅI experiments. The discrepancy between experiments and simulations regarding the values of normal force required to induce a specific indentation depth is due to the different size of the indenters used in experiments and simulations. According to the Hertz contact theory, in a force versus indentation curve, the force scales as the square root of the indenter radius as described in the Experimental Section, see Equations (1) and (2); therefore, a simulated indenter radius ten times smaller than in the experiments translates into forces ≈3 times smaller in the simulations. The atomistic origin of the indentation results reported in Figure 1 is discussed below here.

MD simulations show that upon indentation in a few‐layer thick h‐BN film, the volume in the proximity of the indent undergoes a phase transition to a diamond structure, to an extent that depends on the indentation depth, the indentation contact area, and the thickness of the h‐BN film. As illustrated in Figure 2a,b, the pressure exerted by the simulated indenter promotes a structural and chemical rearrangement of the B–N atoms, which induces in the volume near or underlying the indent a shift from a sp^2^‐hexagonal structure to a sp^3^‐diamond hybridization, with covalent sp^3^ bonds emerging in between the atomic layers. The induced sp^3^‐diamond BN phase includes atoms arranged in both cubic and hexagonal diamond structures. In particular, the cubic phase (c‐BN) accounts for ≈96% of the total new diamond phase, whereas hexagonal diamond represents less than 4%. The percentage and the areal distribution of the converted diamond phase depend primarily on the applied pressure, and the number of layers of the h‐BN film. The four snapshots in Figure 2c show, for the case of a 3L h‐BN film, how pressure activates the sp^2^‐to‐sp^3^ transition, and how the percentage of converted diamond atoms increases with increasing indentation depths. In particular, movies obtained from simulations of the indentation process (see Movie S1 in the Supporting Information) show the nucleation and growth of the metastable sp^3^‐diamond clusters. Pressure affects also the spatial distribution of the sp^3^‐bonded atoms within or near the indented area. As shown in Figure 2d, generally the highest density of converted sp^3^ atoms is not concentrated at the center of the indent, but rather peaks at some radial distance, i.e., at 3 nm for the reported case of 5 Å indentation depth, and *R*_ind_ = 10 nm, for films with L in the range 3−10. Simulations also indicate that the fraction of sp^2^‐to‐sp^3^ converted atoms, namely diamond atoms per layer, is larger in 2L and 3L h‐BN films compared to thicker films, as demonstrated in Figure 2d.

Equipped with the knowledge on the atomistic structural changes occurring during indentation, it is now possible to fully understand the indentation experiments. To this aim, we have conducted a complete quantitative and statistical analysis of the experimental and simulated indentation curves on the h‐BN/SiO_2_ systems, and investigated the effect of the number of layers on the elastic response. **Figure** **3**a displays the effective elastic modulus of h‐BN flakes on SiO_2_ measured as a function of the number of layers via MoNI/ÅI, for a maximal indentation of 5 Å, corresponding to maximal pressures, *P*_max_, in the range 2–4 GPa, depending on the sample stiffness (see Table S1 and Figure S3 in the Supporting Information). The values of the effective elastic moduli, *E*, are extracted by a nonlinear fit of all the experimental MoNI/ÅI curves, similar to the ones shown in Figure 1, using a modified Hertz model (see Equations (1) and (2) in the Experimental Section for further details).^[^ ^15^, ^26^ ^]^ We underline that the extracted effective elastic moduli arise from the deformation of both the h‐BN layers and the underlying SiO_2_ substrate, and represents a combination of their collective elastic responses. The displayed results are data obtained from a total of 93 independent indentation experiments conducted on h‐BN flakes of different thickness. From this analysis, the resulting effective indentation moduli, and respective standard deviations are displayed in Figure 3a. The experimental effective elastic moduli are then compared with the values extracted from the indentation curves calculated by MD simulations for a maximal indentation of 5 Å. The simulated indentation data are then fitted using the same modified Hertz model used for the MoNI/ÅI experiments, and the resulting effective elastic moduli are reported in Figure 3b. Experiments and simulations are in excellent agreement for each h‐BN sample with different number of layers. In particular, they both show that the effective elastic modulus of the bare SiO_2_ substrate and 1L h‐BN on SiO_2_ are the same as expected for the nominal Young modulus of SiO_2_ (≈60 GPa).^[^ ^29^ ^]^ Whereas for 2–3L h‐BN/SiO_2_, E is equal to 94 ± 15 GPa, a 50% increase compared to bare SiO_2_. For 4–5L, E then decreases to 82 ± 8 GPa, and to 40 ± 75 GPa for more than 20L. Indeed, the MD simulations show no sp^2^‐to‐sp^3^ phase transition for 1L h‐BN film, and therefore the effective modulus of the 1L h‐BN/SiO_2_ system is simply the modulus of the bare SiO_2_ substrate, as obtained in experiments and simulations. For the 2–3L h‐BN/SiO_2_ system at about 4 GPa, over 25% of the total atoms within the calculated volume (Figure 2d) undergoes a phase transition to a diamond phase, explaining the observed substantial increase in effective elastic modulus. Regarding the decrease in effective modulus for thicker films observed in experiments and simulations, it is key to consider three factors, namely, the total indentation (5 Å) for the applied local pressures of 2–4 GPa, the low transverse modulus of bulk h‐BN^[^ ^28^ ^]^ (32 GPa), and the strain distribution in the different layers for thicker h‐BN films. **Figure** **4**a,b displays how the total strain distributes between the h‐BN layers and the substrate upon indentation of 5 Å, for different number of atomic layers. The top panel illustrates that for very thin h‐BN films (2L), the substrate is significantly deformed right under the indent, indicating that for this thickness the elastic response of the sample arises from a combination of the stiffnesses of h‐BN and SiO_2_. As the number of h‐BN layers increases, the substrate relative strain decreases, and tends to spread over a larger atomic volume. Looking at the 10L h‐BN sample, we notice that the substrate indentation is considerably reduced, and the pressing indenter deforms predominantly the top h‐BN layers, leaving the h‐BN layers near the substrate less strained. As a consequence, the sp^2^‐to‐sp^3^ phase transition occurs mainly in the top layers, see in Figure S4 (Supporting Information) the percentage of diamond atoms in the different layers for a 10L sample. During a 5 Å indentation, the 10L h‐BN/ SiO_2_ system can be seen as a three‐composites system, with the top layers being a mix of transversely‐soft sp^2^ and stiff sp^3^ phases, the bottom layers being mainly a transversely‐soft sp^2^ phase, and the substrate being SiO_2_. As a result, 10L and ML h‐BN on SiO_2_ are softer than the 2–3L h‐BN flakes on SiO_2_. However, when the indentation depth increases, it is possible to observe a delayed stiffening effect. Indeed, the simulations show that for indentations larger than 6 Å, 10L h‐BN becomes stiffer than bare SiO_2_ (see Figure S5 in the Supporting Information). Finally, we underline that while diamond cubic BN is isotropic, h‐BN is highly anisotropic and therefore has different elastic constants in the different directions. Furthermore, even a completely transformed 2L diamond‐BN film is not elastically isotropic as shown in previous DFT simulations.^[^ ^23^ ^]^ Therefore, the indentation moduli that we are measuring in this study are a result of a complex relationship between the different elastic constants of the partially converted diamond BN films on SiO_2_.

**Figure 3:**
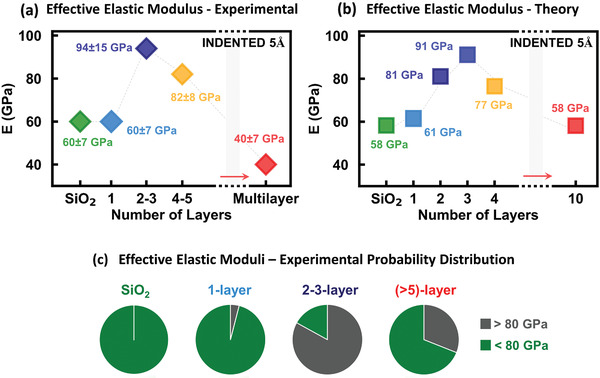
a) Variation of the effective elastic modulus extracted from the MoNI/ÅI experiments on h‐BN flakes, as a function of the atomic thickness, for maximal indentations of 5 Å, corresponding to pressures in the range 2–4 GPa, depending on sample stiffness (see Table S1 in the Supporting Information). b) Variation of the elastic modulus extracted from the MD simulated indentation curves on h‐BN films of different thickness, as a function of the number of layers, for pressures up to 3–4 GPa (Table S1, Supporting Information). c) Probability distribution of the elastic modulus measurements from MoNI/ÅI experiments on h‐BN flakes of different thickness. The gray fraction in the pie charts represents the probability of measuring an elastic modulus higher than 80 GPa.

**Figure 4:**
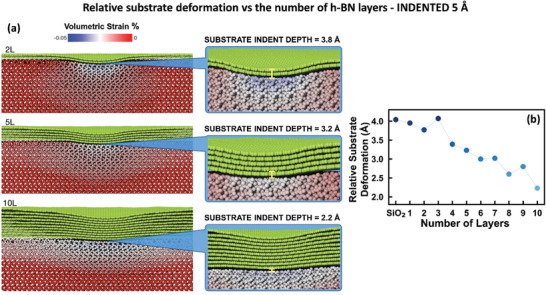
a) Deformation of the h‐BN films + SiO_2_ substrate system for different number of h‐BN layers, i.e., 2‐layer (2L), 5‐layer (5L), and 10‐layer (10L) and for a fix total indentation of 5 Å. Right panels illustrate a zoom‐in of the interfacial area between h‐BN and SiO_2_. b) Relative substrate deformation as shown in (a) as a function of the number of layers in the h‐BN film.

The MD simulations indicate that the pressure induced diamond clusters are metastable (see Movie S1 in the Supporting Information). Therefore, the stiffening phenomenon, being related to the nucleation and growth of the sp^3^‐phase, has a stochastic nature. This can be experimentally observed in the results presented in Figure 3c, where we show the probability of measuring an effective modulus higher than 80 GPa as a function of the increasing number of layers. The value of 80 GPa (20 GPa larger than *E* of SiO_2_) is chosen as a threshold for positive identification of h‐BN flakes having a relevant amount of sp^3^converted atoms. For h‐BN films of 2–3 layers, the number of measurements showing high stiffness is over 80%. On the other hand, while for flakes thicker than 5L the average effective modulus is much lower than 80 GPa, the probability of *E* being larger than 80 GPa remains significantly nonzero, precisely 30%. This result confirms the probabilistic nature of the phase transition, which strictly depends on the amount of strain experienced by the layers in the films. In the case of multilayer structures (>20 layers), the probability of observing high stiffness sharply decreases to 0%. The pressures (2–4 GPa) identified to activate the phase transition to a diamond‐BN phase are significantly smaller than those generally observed in h‐BN hydrostatically compressed during diamond anvil cells (DAC) tests.^[^ ^21^ ^]^ In general, nonuniform strain fields and shear stresses have proven to lower the critical pressure of the sp^2^‐to‐sp^3^ transitions in 2D materials.^[^ ^21^, ^30^ ^]^ The uniaxial indentation exerted during the MoNI/ÅI experiments, coupled with the extra strain caused by the deformation of the substrate, generates local shear stresses and a nonuniform stress field among the h‐BN layers that favor the local lattice distortion and therefore lower the pressures necessary to locally induce the atomic rearrangement to a sp^3^ phase. The pressures proposed in this work are in the similar order of magnitude as experimental data available in literature for nonuniform, compression‐induced sp^2^‐to‐sp^3^ phase transition of h‐BN crystal.^[^ ^21^, ^24^ ^]^

KPFM experiments are conducted in parallel with Raman spectroscopy to further characterize the nature of the pressure‐induced phase transition in the h‐BN flakes. A complete description of the experimental procedure is included in the Experimental Section. **Figure** **5**a displays the contact potential measured on a h‐BN flake before and after scanning the h‐BN films with an AFM probe while applying a contact load of ≈300 nN. Notably, after the pressure is uniformly applied to the scanned area, the contact potential of the h‐BN flake remains approximately constant everywhere on the sample surface except on the 2–3L h‐BN flake, where it increases to approximately 50 mV. A change in KPFM contact potential upon compression has been previously reported in Ref. [24]; however, our results differ in terms of values, possibly due to the humid environment in which the experiments in Ref. [24] have been conducted. Since the variation in the local contact potential upon compression can indicate the modification of the lattice structure in 2–3L h‐BN, and the transition to a different crystal phase, we use Raman spectroscopy to investigate the film structure before and after pressurization. By recording the KPFM contact potential from the pressurized area over time, we observe that the pressure‐induced change in the surface potential and the consequent structural modification persist for more than 30 min after the release of pressure (see Figure S6 in the Supporting Information). Figure 5b displays two regions of the Raman spectrum that are significant for the characterization of BN films, namely, the interval between 1300 and 1400 cm^−1^ – right panel – where the characteristic peak associated to the in‐plane vibration of the h‐BN layers is located, and the region between 1040 and 1060 cm^−1^ – left panel – which is the region of the two characteristic peaks of cubic boron nitride (c‐BN).^[^ ^31^ ^]^ The Raman spectra reported in Figure 5b are acquired from the 2–3L h‐BN region of Figure 5a before (gray line) and after the compression (blue color scale lines). By using the prepressure spectrum (light gray) as a reference, we can observe in the spectrum acquired immediately after compression (labeled as +0′, dark‐blue) the emergence of an additional peak at 1048 cm^−1^ that is associated to the transverse optical phonon mode of c‐BN.^[^ ^32^ ^]^ In agreement with the KPFM results of Figure S5 (Supporting Information), the structural transformation persists even minutes after the compression (+7′, dark‐blue). The c‐BN peak eventually disappears after ≈55 min (+55′, light‐blue), with the Raman spectrum reverting to the initial prepressure condition. The low intensity of the c‐BN longitudinal optical mode peak at 1304 cm^−1^, around 30% weaker than the transverse mode peak,^[^ ^32^ ^]^ prevents it from being detected. Whereas only based on KPFM and Raman evidence we cannot entirely exclude the formation of other hard BN phases such as wurtzite BN,^[^ ^23^ ^]^ the Raman peak found at 1048 cm^−1^ strongly suggests the presence of the c‐BN phase. Figure 5b shows the variation of the intensity of the h‐BN characteristic peak (at 1369 cm^−1^) after compression. The intensity of this peak is reduced significantly immediately after compression (0′ and 3′) due to the rearrangement of part of the hexagonal lattice into a cubic structure. However, as time goes on, this peak recovers the initial prepressure intensity, indicating a relaxation of the local structure to the initial hexagonal configuration.

**Figure 5:**
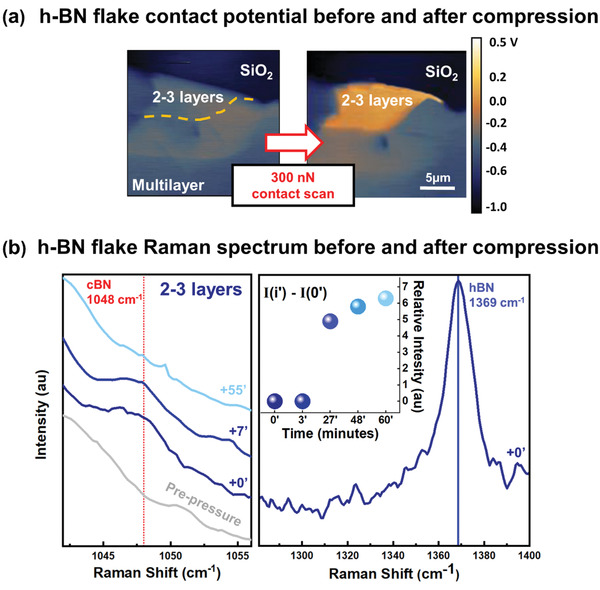
a) KPFM map of a h‐BN flake including 2–3L and multilayer h‐BN areas, before (left panel) and after (right panel) compressing the surface using a contact load of ≈300 nN. b) Left panel: Raman spectrum acquired on the 2–3L h‐BN area of the flake displayed in (a), before (gray spectrum) and at different point in time after compression of the surface (blue‐scale spectra). The red dotted line highlights the spectral position of the characteristic c‐BN structure Raman peak. Right panel: Raman spectrum acquired on the 2–3L h‐BN area immediately (0 min) after compression, highlighting the Raman frequency region where the h‐BN characteristic peak is located (≈1369 cm^−1^). The scale of the *y*‐axis intensity is the same for Left and Right panel. Inset: relative intensities of the h‐BN peak acquired at different time steps after compression. The intensities reported are relative to that recorded for the spectrum at 0 min after compression.

In summary, we demonstrate that at room temperature and with no surface chemical functionalization, local pressure in atomically thin h‐BN on a SiO_2_ substrate activates the formation of a diamond BN phase, through sp^2^‐to‐sp^3^ rehybridization and structural atomic rearrangement. MD simulations show a metastable local rearrangement of the h‐BN atoms into diamond crystal clusters within and around the indentation area, when increasing the indentation pressure. Raman spectroscopy experiments confirm the presence of a pressure‐induced cubic BN phase, and prove its metastability over time upon release of pressure. Furthermore, MoNI/Å‐indentation experiments and MD indentation simulations show that at pressures of 2–4 GPa, the formation of a pressure‐induced diamond BN phase entails an increase in indentation stiffness in 2–3L h‐BN on SiO_2_ of up to 50% compared to the value of the bare SiO_2_ substrate, and of more than 300% compared to the value of the out‐of‐plane elastic modulus of bulk h‐BN, i.e., ≈32 GPa,^[^ ^28^ ^]^ However, when increasing the number of layers, at similar maximal indentation pressures of 2–4 GPa, the h‐BN/SiO_2_ system becomes softer and softer, reaching the value of 40 GPa for more than 20 layers. The simulations show that even for thicker films the top layers undergo a sp^2^‐to‐sp^3^ phase transition; however, the softening can be understood in terms of a reduced strain for pressures up to 4 GPa in the layers closer to the substrate, decreasing the probability of the diamond phase formation, and thus leaving the underlying layers softer in the out‐of‐plane direction. In conclusion, this work offers a new understanding of the pressure induced phase transition of atomically thin h‐BN into 2D diamond BN, and opens up new avenues for the mild temperature synthesis of 2D diamond BN. The results presented here also shed light on the potential and benefits of using atomically thin h‐BN films in a variety of applications that require excellent performances under localized pressures, such as protective ultra‐thin coatings, composites reinforcement, and films for opto‐mechanical modulation.

## Experimental Section

##### Preparation of Exfoliated h‐BN on SiO_2_

Exfoliation of h‐BN was performed starting from bulk crystals provided by HQ Graphene. The procedure performed for exfoliation is similar to the one described in Ref. [7]. Several large areas few‐layer h‐BN flakes were isolated at each synthesis. Excess residual glue was removed by heating the substrate to 350–400 °C in vacuum for 1 h. The thickness of the flakes was initially assessed using optical microscopy, while the exact number h‐BN layers was estimated using AFM imaging on a Bruker Multimode microscope. More details are presented in Figure S1 in the Supporting Information.

##### MoNI/Å‐Indentation for Transverse Elasticity of h‐BN Flakes

MoNI/Å‐indentation is discussed in details in Refs. ^[^ ^15^, ^26^, ^27^ ^]^. For the experiments presented here, a sinusoidal voltage (<0.6 mV) applied using a lock‐in amplifier (Stanford Research Systems, SR830) to the piezotube of a commercial AFM (Agilent PicoPlus AFM) was used to drive small oscillations (Δ*z*_piezo_ ≈ 0.1 Å) in parallel to the main AFM driving voltages. During indentation, a polycrystalline diamond‐coated silicon AFM indenter (Nanosensors DT‐NCHR, with spring constant ≈ 80–100 N m^−1^, radius ≈ 100 nm) was brought into contact with the h‐BN flake at the desired maximum normal load, corresponding to the maximum pressure to apply. The indenter was then slowly retracted from the sample, until it completely lost contact with the surface. The force versus indentation depth curves were acquired during this unloading phase. The indentations were performed by adopting a point scan mode, whereby different locations on the flake and on the substrate were assessed via AFM topography, and tested during the same set of experiments, allowing direct comparison of indentation curves measured on h‐BN and on SiO_2_. The indentation modulus of h‐BN on SiO_2_ was estimated by fitting the force *F_z_* versus indentation *z*_indent_ curves with the modified Hertz (DMT) model^[^ ^33^ ^]^

(1) $$\begin{array}{c} F_{z}=\frac{4E^{*}\sqrt{R}}{3}\;z_{\mathrm{indent}}^{3/2} \end{array}$$

using the indentation modulus E* as the fitting parameter. Here *R* is the radius of the indenter. More information are available in the Supporting Information of Ref. [34]. The effect of the adhesive forces was not neglected during the MoNI/ÅI experiments. Indeed, *F*_po_ is the pull‐out force measured by the AFM when the tip loses contact with the sample's surface, and it is therefore equivalent to the adhesion force. The indentation curves are hence obtained considering the corrected “absolute” normal load *F_z_*  =  *F_z_* − *F*_po_ in Equation (1). The effective elastic modulus of the h‐BN/SiO_2_ system is computed using the following formula

(2) $$\begin{array}{c} E=\frac{1-\nu^{2}}{\frac{1}{E^{*}}-\frac{1-\nu_{\mathrm{ind}}^{2}}{E_{\mathrm{ind}}}} \end{array}$$

where E, and *ν* are the effective elastic modulus and Poisson's ratio of the surface and *E*_ind_, *ν*_ind_ are the modulus and Poisson's ratio of the AFM indenter. Here, *ν* = 0.2 was used for h‐BN,^[^ ^1^ ^]^ *E*_ind_ = 1050 GPa and *ν*_ind_ = 0.2 for the indenter.^[^ ^26^ ^]^ The extracted *E* is defined as the effective elastic modulus of the h‐BN/ SiO_2_ system, as it represents the combination of the elastic response of both the h‐BN layers and the underlying substrate. Pressures applied during the MoNI/ÅI experiments were estimated to be in the range 2–4 GPa.

##### MD Simulation

To simulate the nanoindentation of the h‐BN thin films the open‐source molecular dynamics simulation code LAMMPS^[^ ^35^ ^]^ (Large‐scale Atomic/ Molecular Massively Parallel Simulator) was used. The Extended Tersoff Potential (ExTeP) developed by Los et al.^[^ ^36^ ^]^ was employed to describe the interatomic interactions in the h‐BN layers. For the interactions between the h‐BN thin film and the substrate, the associated cross‐potential interactions were described between B–Si, B–O, N–Si, and N–O by employing a standard 12/6 Lennard–Jones potentials as presented in Ref. [37]. A more detailed description of the modeling methodology is presented in the Supporting Information.

##### KPFM and Raman Spectroscopy Experiments

KPFM and Raman spectroscopy was conducted in parallel to assess the effect of pressure on the structural transformation of the h‐BN flakes. Frequency modulated KPFM (FM‐KPFM) experiments were performed with a Bruker Multimode 8 AFM, using a Pt/Ir coated indenter (PPP‐EFM Nanosensors, with spring constant ≈3 N m^−1^ and indenter radius ≈25 nm). To apply the pressure on the h‐BN layers, a normal load of ≈300 nN was applied to the flake and the surrounding substrate while scanning the surface in contact mode with the same Pt/Ir coated indenter. FM‐KPFM maps were recorded before and after the pressure was applied to the surface, to check for modification in the surface contact potential difference. Raman spectra were acquired in the region of the KPFM scan using a Horiba Scientific LabRam HR Evolution confocal Raman system, equipped with an objective lens 100x, NA = 0.8. A laser line excitation at *λ* = 532 nm was used. Raman experiments were conducted before and after the pressure was applied to the flake using the AFM.

## Conflict of Interest

The authors declare no conflict of interest.

## Supporting information

Supporting Information
