# Supplementary material for: Pressure‐Induced Formation and Mechanical Properties of 2D Diamond Boron Nitride
Source: Adv Sci (Weinh). 2020 Dec 11;8(2):2002541. doi: 10.1002/advs.202002541 (PMC7816702; doi:10.1002/advs.202002541)
Supplement: Supplementary file 1 — Supporting Information [file ADVS-8-2002541-s001.pdf]

© 2020 Wiley-VCH GmbH

ADVANCED SCIENCE Open Access

Supporting Information

for *Adv. Sci.*, DOI: 10.1002/advs.202002541

Pressure-induced Formation and Mechanical Properties of 2D Diamond Boron Nitride

*Filippo Cellini[1,†], Francesco Lavini[1,2,†], Elton Chen[3], Angelo Bongiorno[4,5], Filip Popovic[1], Ryan L. Hartman[1], Remi Dingreville[3], and Elisa Riedo[1]**

Supporting Information

Pressure-induced Formation and Mechanical Properties of 2D Diamond Boron Nitride

Filippo Cellini[1,†], Francesco Lavini[1,2,†], Elton Chen[3], Angelo Bongiorno[4,5], Filip Popovic[1], Ryan L. Hartman[1], Remi Dingreville[3], and Elisa Riedo[1]*

[1] Tandon School of Engineering, New York University, Brooklyn, NY, 11201, USA

[2] Department of Physics, New York University, New York, New York 10003, USA

[3] Center for Integrated Nanotechnologies, Sandia National Laboratories, Albuquerque, NM 87185, USA

[4] Department of Chemistry, College of Staten Island, City University of New York, Staten Island, NY 10314, USA

[5] CUNY Graduate Center, Ph.D. Program in Chemistry, New York, NY 10016, USA

* Corresponding author**:** elisa.riedo@nyu.edu

† These authors contribute equally to this work.

Optical micrographs, topography, and phase imaging of exfoliated h-BN flakes. Exfoliated h-BN samples are prepared following the procedures described in the Experimental Section of the main manuscript. The structure of the flakes, and in particular the number of layers in each region of the flake, is assessed using optical microscopy and AFM imaging. An example of the analysis performed is reported in Figure S1: a region of the flake showing optical hue and contrast associated to few layers h-BN is identified from the optical image in Figure S1(a), see Reference[1] for details on the optical identification of 2D layers in exfoliated flakes; surface topography and phase images are measured in AFM tapping mode, as shown respectively in Figure S1(b) and (c), in order to identify the number of layers.

Figure S1(b) displays the AFM topography of the region indicated with the dashed square in the optical micrograph in Figure S1(a). Topography shows the typical landscape observed in 2D films, with sharp edges indicating edges of 2D crystals of different thickness. Profiles are analyzed using post-processing tools to assess the exact thickness of the flake (see Profile A and B in Figure S1). 2D film thickness is directly correlated with the effective number or layers by comparing experimental data with the nominal thickness of h-BN layers (~0.33 nm). Moving left to right in the topography graph, Profile A shows an initial step of approximately 0.4 nm from the SiO_2 substrate to monolayer h-BN, and a second step of approximately 0.5 to 0.7 nm from monolayer film to the 2-3-layer h-BN film. A similar analysis is conducted on Profile B, whereby h-BN monolayer is identified in the topography between the 2-3-layer and the 4-layer regions. An identical approach is applied to the flake presented in Figure S2, where it is possible to distinguish between a 2-layer and a 3-layer h-BN regions thanks to a precisely discernable difference in their topographic profiles.

Figure S1(c) displays the AFM phase shift image measured during scanning of the flake in tapping mode. To the best of our knowledge, contrast in AFM phase images does not provide a clear indication on the effective number of h-BN layers. However, if properly correlated with topography and optical microscopy, information from phase images can help identify areas of the scan that are part of the h-BN flake and the SiO_2 substrate. Specifically, in Figure 1(c), the area in the top left region of the scan showing higher phase shift is univocally associated to the SiO_2 substrate, as confirmed from the optical micrograph in Figure S1(a).

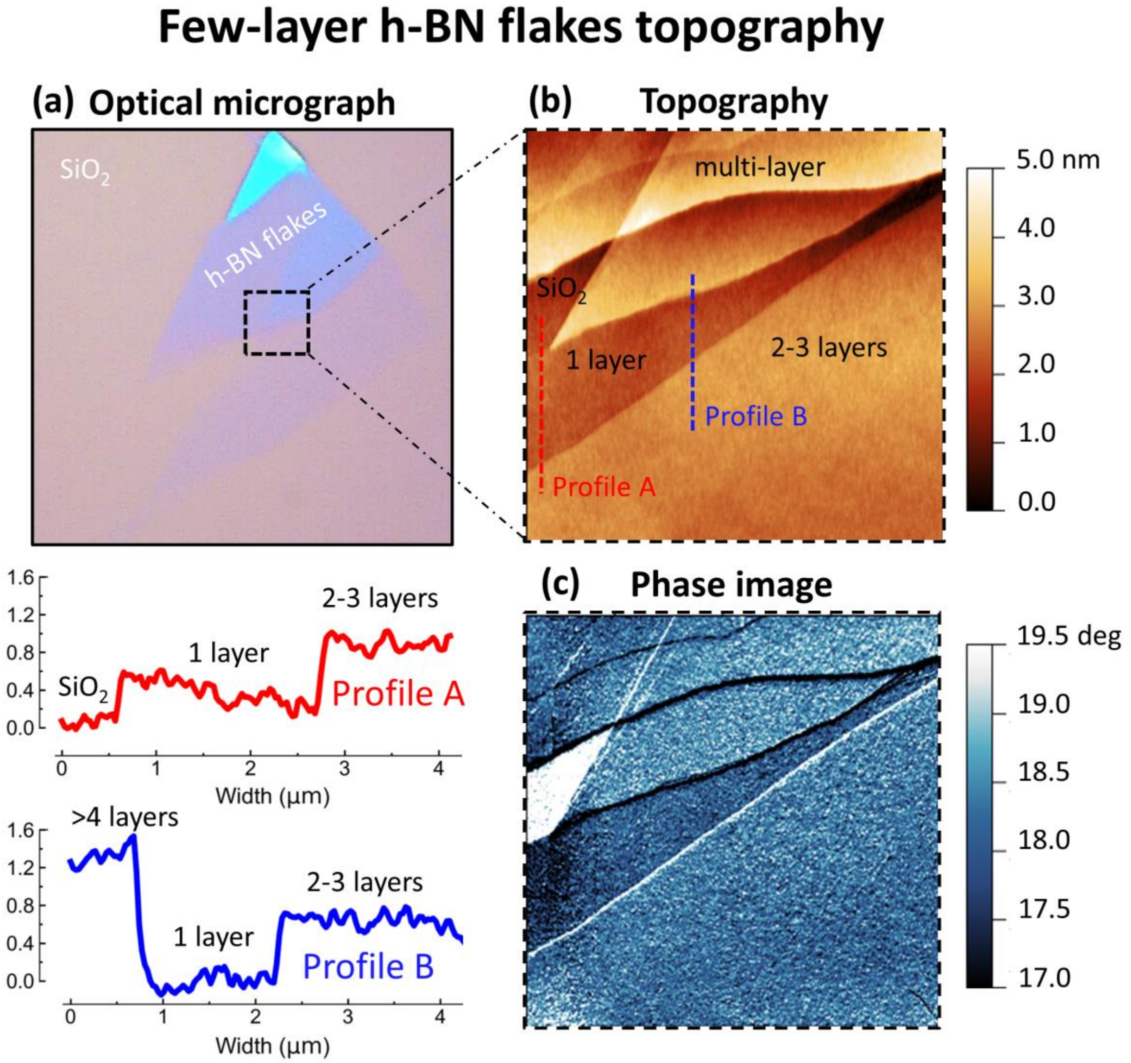

Figure S1. a) Optical micrograph of exfoliated h-BN flakes on SiO_2, with areas of thickness ranging from single-layer to multilayer (> 50) h-BN. b) Tapping mode AFM topography of the flake area in (a) demarked with a black dashed square. The red and blue dashed lines indicate the position where the Profile A and Profile B where acquired, respectively. The profiles show h-BN areas of different thickness and illustrate how AFM topography is used to assess flakes' morphology and thickness. (c) AFM phase imaging corresponding to the topography reported in (b).

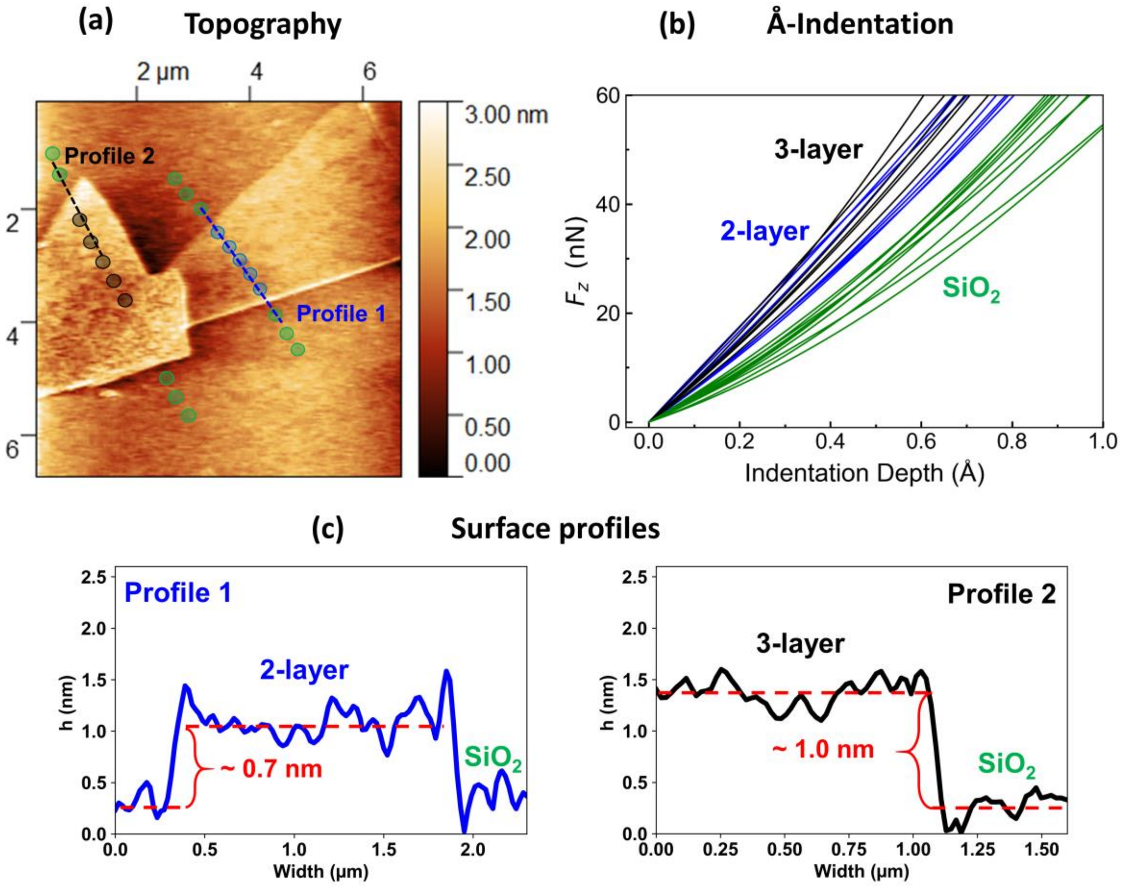

Figure S2. a) AFM tapping mode topography of a h-BN flake on SiO_2. b) Force versus indentation depth curves obtained via Å-indentation in the positions highlighted by marker points in (a), respectively for 2-layer h-BN (blue), 3-layer h-BN (black), and the SiO_2 substrate (green). c) Topography profiles extracted from the dotted lines in (a), showing respectively a 2-layer h-BN region (blue – Profile 1) and a 3-layer h-BN region (black – Profile 2).

Supplementary Movie 1 – ***Evolution of the amount of transformed diamond-BN in a 3-L h-BN film, with increasing indentation depth.***

Refer to the included movie:

Supplementary Movie 1 - Phase_Transformation_3L h-BN

Supplementary Table S1. Value of the maximum pressure used during MoNI/ÅI experiments, corresponding to indentation of 5 Å, for h-BN flakes of different thickness. The indentation radius is calculated following the Hertz model for a sphere in contact with a planar surface[2], using the average elastic moduli E reported in Fig.3(a) of the main text.

Max pressure P						
# Layers-film	E(GPa)	E*(GPa)	F_{max} (nN)	z_{ind} (Å)	Indent radius (nm)	P_{max} (GPa)
Si02	60	59.1	278.7	5	7.1	2.7
1L	60	59.1	278.7	5	7.1	2.7
2-3L	94	89.9	423.7	5	7.1	4.0
4-5L	82	79.2	373.5	5	7.1	3.6
ML	40	40.1	201.8	5	7.1	1.8

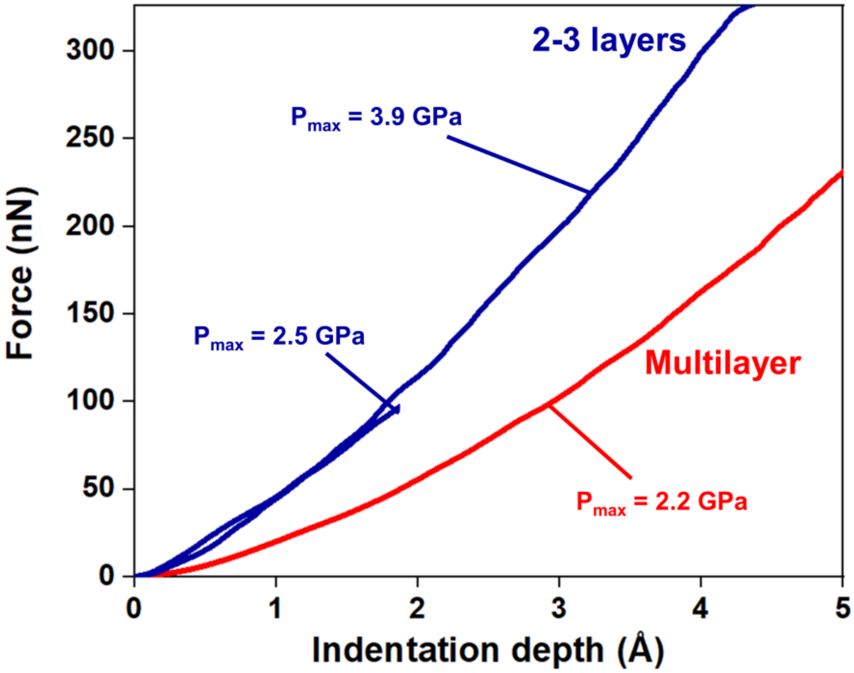

Figure S3. MoNI/ÅI curves performed at different maximum pressure on 2-3L h-BN and on ML h-BN flake. We can see that in the range of 2-4 GPa considered in the main manuscript, no relevant variation is observed in the stiffness of the 2-3L h-BN curves. The experimental conditions achieved during the indentations are reported in the table below:

Max pressure P					
# Layers-film	E(GPa)	F_{max} (nN)	z_{ind} (Å)	Indent radius (nm)	P_{max} (GPa)
2-3L (1)	96.0	356.6	4.4	6.6	3.9
2-3L (2)	101.0	97.7	1.8	4.2	2.5
ML	47.0	235.6	5.2	7.2	2.2

Percentage of converted diamond-BN atoms in 3-layer, 4-layer and 10-layer h-BN film. Figure S4 reports the percentage (%) of converted diamond-BN atoms in each layer composing the 3-layer, 4-layer and 10-layer h-BN film, integrated over the indentation depth. Each value (per each indentation depth data point) is obtained as the ratio between the number of converted diamond-BN atoms divided by the total number of atoms within the indent area. We can see that in the 10-layer film, the bottom-most layers (7L, 8L, 9L and 10L) show low percentages of converted diamond-BN. Some of the total final percentages have values above 100% as we are not considering the diamond atoms that are revert to the hexagonal phase. Indeed, we can see from Fig. 2c in the main manuscript that a fraction of atoms transformed to the diamond phase at low indentation may revert to the hexagonal phase later at later stages of the indentation.

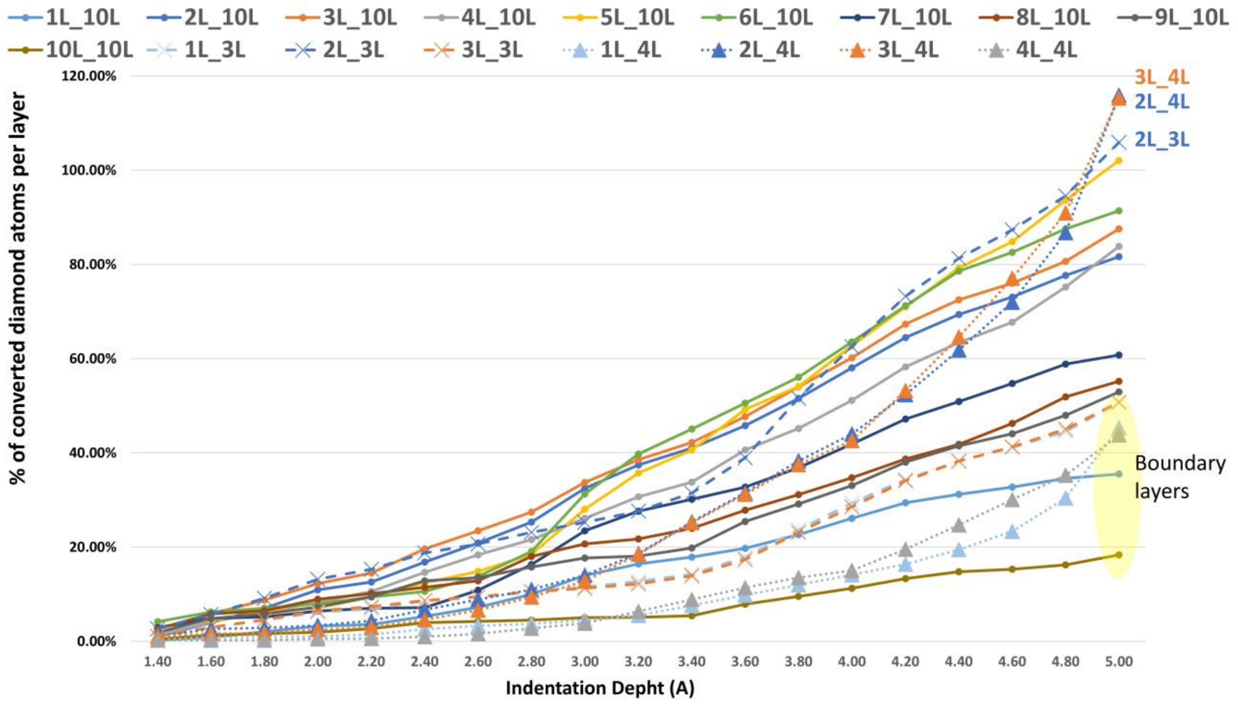

Figure S4. Percentage (%) of converted diamond-BN atoms in each layer composing the 3-layer, 4-layer and 10-layer h-BN film, integrated over the indentation depths. Regarding the legend, the first part of the label indicates the layer number (n-th layer), while the second indicates the h-BN film. So, for example, "4L_10L" indicates the 4-th h-BN layer in the 10-layer film. Boundary layers are the layers that are either in contact with the substrate (the bottom-most layer of the film, i.e. 3L_3L, 4L_4L and 10L_10L) or the topmost layer (the first h-BN layer, i.e. 1L_3L, 1L_4L and 1L_10L).

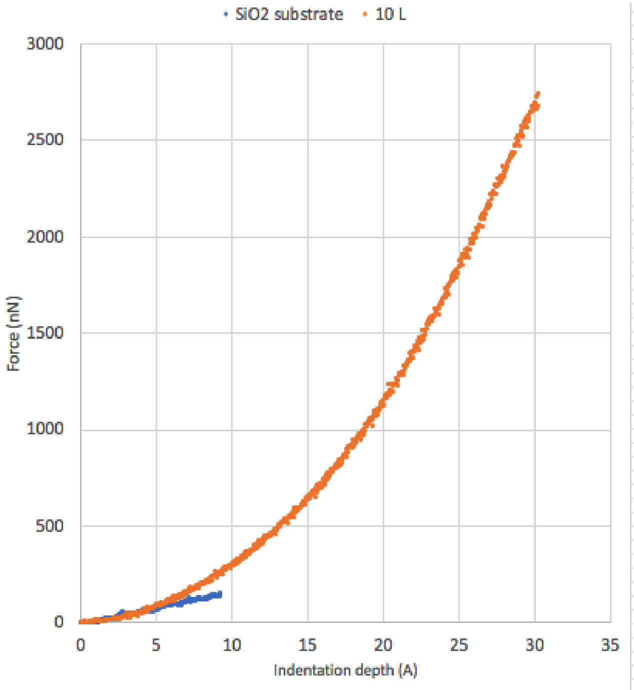

Figure S5. Force-indentation curves extracted from the MD indentations calculated on 10-layer h-BN film and SiO_2 substrate, for indentation depths larger than 5 Å. As reported in the main manuscript, the 10L h-BN film appears to be softer than SiO_2 for small indentation depths. However, when the force and the indentation depths increase, the strain distributes through all the 10 layers, inducing higher percentages of diamond-BN phase even in the bottom-most layers, and causing an increase of the overall film stiffness.

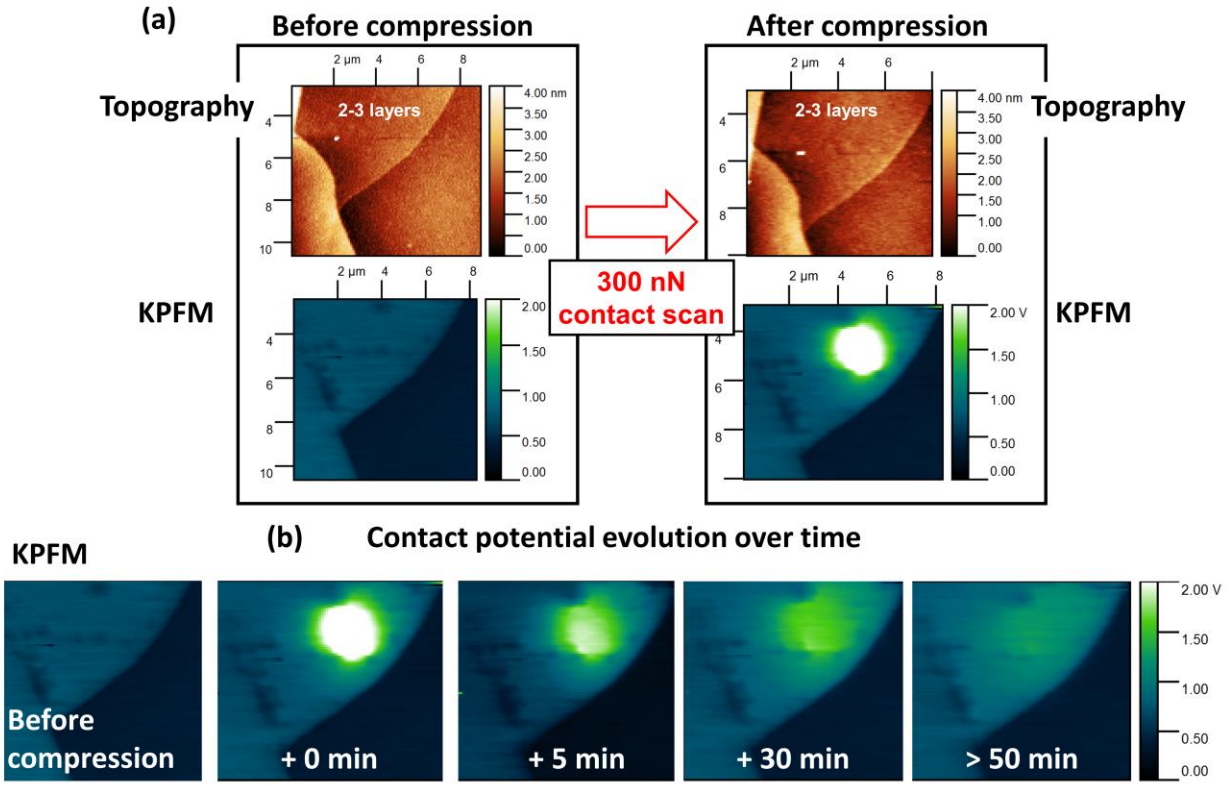

Figure S6. a) AFM topography and KPFM image of a 2-3-layer h-BN flake, before (left panel) and after (right panel) scanning the surface with high contact load (~ 300 nN). b) Evolution of the KPFM contact potential over time. The contact potential is monitored from the surface area of (a) at different time points before (first panel from the left) and after the compression.

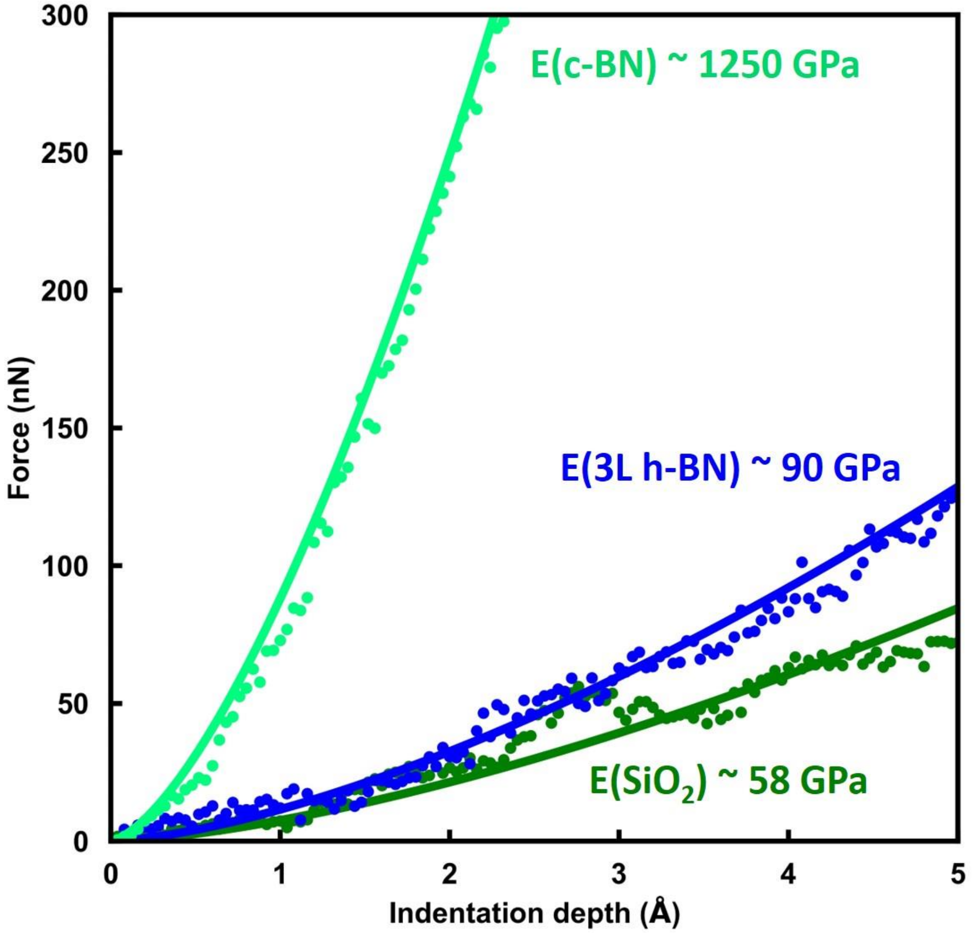

Figure S7. Comparison between the force-indentation curves extracted from the MD indentations calculated on the 3-layer (3L) h-BN film on SiO_2, on bare SiO_2 and on bulk c-BN, with the respective elastic moduli (E). As explained in the main manuscript, the effective elastic modulus extracted on 3L h-BN is a combination of the elastic response of the h-BN layers, undergoing the stiffening phase transition to diamond-BN, and the underlying substrate. For films this thin, and indentation depths this small, it is quite complex, even computationally, to extract the indentation elastic modulus of the sole diamond BN films, ruling out completely the contribution of the substrate. We can only conclude that the indentation elastic modulus of the pressure transformed 3L diamond-BN films is lower than the indentation modulus of isotropic bulk c-BN (~ 1250 GPa) and larger than the measured modulus (90 GPa) of the system composed of the 3L BN film on the SiO_2 substrate (60 GPa).

Molecular Dynamics (MD) simulation modeling methodology. As explained in the Experimental Section of the main manuscript, we use the open-source molecular dynamics simulation code LAMMPS[3] to simulate nanoindentation of the hBN thin films, and the Extended Tersoff Potential (ExTeP)[4] to describe the interatomic interactions in the h-BN layers. To model the SiO_2 substrate, we use the Tersoff potential developed by Munetoh et al.[5]. For our

model, we assume a predominant alpha Quartz crystal structure that does not undergo phase change during indentation. For the interactions between the hBN thin film and the substrate, we describe the associated cross-potential interactions between B-Si, B-O, N-Si, and N-O by employing a standard 12/6 Lennard-Jones potentials as presented by Ni et al.[6].

The thin-film/substrate system is created by first constructing the SiO_2 substrate as a 40 x 60 x 20 superlattice comprising 648,000 atoms and approximate 304 x 264 x 110 $Å^3$ in dimension. Surface h-BN layer(s) are then added in the normal Z-direction with each layer consisting of 28,800 atoms. The number of layers simulated ranged from one layer to ten layers. The thin-film/substrate system is constructed with periodic boundary conditions in the X-Y directions while minimizing the misfit strain in the lateral directions. Such boundary conditions mimic a thin-film resting on a substrate. After constructing the thin-film/substrate system at 0K, the simulation cell is equilibrated by minimizing the energy of the system and then thermalized to 300K using a isobaric-isothermal (NPT) ensemble at zero pressure and at 300K for 20 ps using a fixed 1 fs time step. This procedure provided the minimum energy configuration and relaxed configuration for the thin-film/substrate system.

In the indentation simulations an artificial rigid spherical indenter with radius of 100 Å is introduced at 100 Å above the surface layer(s) moving at a constant velocity of 0.2 Å /ps towards the film. The force exerted by the indenter follows the Equation $F(r) = -K(r - R)^2$, with R being the radius of the indenter, r being the distance from the indent center, and K designated as the force constant of 10 eV/ $Å^3$. To allow free deformation of the surface layer(s) and the substrate, the simulations are performed in a microcanonical ensemble (NVE ensemble, i.e., isovolumetric, isoenergetic). In addition, the atoms at the bottom 10 Å of the substrate are fixed as static in order to prevent the acceleration and movement of the bulk systems. The zero-indentation depth is calculated as the earliest indenter displacement where significant forces (> 5 nN) are consistently evaluated under the indent.

References

[1]P. Blake, E. Hill, A. Castro Neto, K. Novoselov, D. Jiang, R. Yang, T. Booth, A. Geim, Making graphene visible, *Appl Phys Lett*, 91 (**2007**) 063124.

[2]U.D. Schwarz, A generalized analytical model for the elastic deformation of an adhesive contact between a sphere and a flat surface, *Journal of Colloid and Interface Science*, 261 (**2003**) 99-106.

[3]B. Hendrickson, S. Plimpton, Parallel many-body simulations without all-to-all communication, *Journal of Parallel and Distributed Computing*, 27 (**1995**) 15-25.

[4]J. Los, J. Kroes, K. Albe, R. Gordillo, M. Katsnelson, A. Fasolino, Extended Tersoff potential for boron nitride: Energetics and elastic properties of pristine and defective h-BN, *Phys Rev B*, 96 (**2017**) 184108.

[5]S. Munetoh, T. Motooka, K. Moriguchi, A. Shintani, Interatomic potential for Si–O systems using Tersoff parameterization, *Computational Materials Science*, 39 (**2007**) 334-339.

[6]Y. Ni, J. Jiang, E. Meletis, T. Dumitrică, Thermal transport across few-layer boron nitride encased by silica, *Appl Phys Lett*, 107 (**2015**) 031603.
